# Evaluating the Impact and Potential Impact of Machine Learning on Medical Decision Making

**DOI:** 10.1177/0272989X221146506

**Published:** 2022-12-28

**Authors:** Lauren E. Cipriano

**Affiliations:** Deputy Editor, *Medical Decision Making* and *MDM Policy & Practice*; Ivey Business School and Departments of Medicine and Epidemiology and Biostatistics, Schulich School of Medicine & Dentistry, Western University, London, ON, Canada

The best predictor of future behavior is past behavior.

*—Attributed to many authors*

At the recent Society for Medical Decision Making annual meeting, keynote speaker Dr. Erich Huang advised us to “think about machine learning, algorithms we generate from data, etc. as extensions, not replacements” of humans. He reminded us that the use of artificial intelligence (AI) does not obviate our professional responsibilities for patient-centered and patient values–guided decision making in health policy and medicine.

Integrating AI into health system operations and clinical decision making promises a more effective, efficient, and personalized health care system. However, there are unresolved limitations of the science and operational challenges, leading to numerous high-profile examples in which the technology grossly fails to achieve its promise. As for other tools of decision science, the context, setting, data, role of uncertainty, and human interaction with the information are key factors for realizing success. As such, there is a substantial role for the medical decision-making research community to contribute to the effective design and use of AI tools in health care and medicine.

## What Is Machine Learning?

Machine learning (ML) refers to a set of analytical techniques, algorithms, and mathematical models used for automated and dynamic prediction within the field of AI. As a field, AI aims to develop computerized systems that behave in ways that mimic (and, perhaps, exceed) human capabilities. AI-enabled programs analyze data to provide information that can support decision making or automatically trigger actions without human intervention.

## ML Tools Create Dynamic Predictive Models

Relying on many of the same tools and algorithms as statistics, ML emphasizes prediction in high-dimensional problems. What differentiates ML approaches from standard statistical models used for prediction in medicine is that ML can be dynamic, such that the data features used for prediction, the way in which those features influence the prediction (e.g., in regression, the beta coefficients), and even the model structure itself update as new data come in, relying on automated model selection and fitting procedures. ML lends itself to a broad set of tools, including those that make fewer assumptions than classic statistical approaches, often leveraging nonparametric or semiparametric models and nonlinear relationships, and foregoing the benefits and interpretability of statistical inference.^1^

While it is possible, within an ML framework, to use a single model structure (e.g., linear or logistic regression, regularized regression, classification trees, support vector regression, k-means clustering, etc.), it is also possible to use an ensemble of methods in which different combinations of methods are used over different regions of the predictor or predicted space. ML also includes a set of tools characterized as “deep learning,” which represent artificial neural networks in which probabilistic prediction occurs through multiple layers of mathematical modeling based on a network structure. Selection of the best model is identified using automated approaches to model selection, iteratively partitioning the data set, within-sample fitting, and out-of-sample evaluation. The model selection criterion seeks to minimize prediction error on out-of-sample data.

With the primary focus on predictive accuracy, some design features essential to interpretability of hypothesis tests, such as independence of predictors to avoid multicollinearity, are no longer required. As a result, interpretability and inference related to the fitted model coefficients, even when using methods for which they exist, is generally lost. Many model structures used in ML have no interpretable coefficients or weights. Evaluating face validity of ML models and identification of influential factors often requires exploration of small perturbations in the input parameters and the use of counterfactual analysis.^2^

Exacerbating the black-box nature of ML tools is the dynamic nature of the model structure and of the coefficients or weights placed on various predictive features. As the data set adds new observations over time, the automated algorithm uses the new data to improve the prediction model (read: the machine learns). As a result, a decision support system with an ML engine may not make the same recommendations for the same decision problem at different points in time. In many ways, this recommendation update resembles a physician who has, in the interim, attended a training session and learned to recommend something new. However, without explanation, users may find the change of recommendation disconcerting, confusing, and untrustworthy.

## Challenges of Implementing ML-Based Prediction in Practice

The dynamic black-box nature of ML tools makes the validity of the resulting models challenging to assess. After implementation, a model may drift in response to the new data, creating an ongoing challenge of model evaluation.^3^

The data used in ML tools are generally observational in nature, and the resulting prediction analysis has all the classic limitations of observational data: selection bias, patient features that may be predictive of both treatment choice and outcome, imbalance in the data in the predictor space, unobserved confounding, and so forth.^4^ When the goal of ML-based prediction is to inform a decision, there is an implicit assumption of causality related to that decision problem, which may not be correct as ML approaches are challenged by the same issues as traditional statistical approaches when using observational data.^5^

Structural and systemic biases that exist within the system in which the data are collected will be present in the data as unobserved confounders.^6–8^ Famously, ML-driven tools mirror the structural racism and cultural bias of the system from which the data are sourced.^9–11^ Further, in the automated model selection process, the overall quality of model prediction may be achieved by sacrificing the quality of prediction for marginalized persons and other underrepresented minority groups in exchange for superior quality of prediction for majority groups.^12^

Ultimately, the use of an ML tool to direct decisions may create homogeneity in decisions among similar patients, and as those patients become part of the data set for future predictions, they may enrich the data set in unbalanced and potentially self-fulfilling ways.^13^ This occurs because the outcomes of decisions become part of the data, compounding selection bias and imbalance in the predictor space. Data points representing alternative decisions for similar-looking patients may not be explained within the model framework because there may be unmeasured reasons that the patient and physician chose not to follow the ML-derived recommendation. Health administrative systems are particularly at risk of missing variable bias because they do not contain many variables known to have causal influence on patient outcomes, including many social determinants of health.

Another limitation of all models that rely on observational data is the use of proxy variables for unmeasured variables. When prediction accuracy is of paramount importance, the use of proxy variables is not generally considered problematic. However, in the context of many applications in social science and medicine, and in the setting of decision support, it may be highly problematic if the proxy variable is masking patient characteristics that are generally deemed unethical dimensions on which to base clinical recommendations (e.g., race^11,12^).

Furthermore, the lack of transparency in predictors can mask opportunities to improve patient outcomes. Key causative variables can fail to be identified. Access and use of allied health support, such as rehabilitation after stroke through supplemental insurance, may be what actually differentiates patient outcomes, but the proxy of zip code (a proxy itself for wealth) may be identified as a predictor when access to these services is unrecorded. Poor-quality proxy variables that do get selected into predictive models may aggravate existing challenges in diagnosis and treatment decisions because they engrain stereotypes into the algorithm.^7^

More difficult to detect because of the black-box nature of ML models, system-level confounders and missing variables lead to a situation in which models are not generalizable across settings. The same patient presenting to a hospital in a low-income neighborhood may get a different recommendation than if they present to a hospital in a high-income neighborhood, in part because which hospital a person goes to is predetermined by numerous factors. As a result, model validation using standard best practice—validation against an independent data set—is a challenge and, perhaps, not applicable. At the same time, this situation should remind us that the important question is not how the algorithm can be fixed to work effectively in both settings. Instead, the right question is how we can use the many tools at our disposal, including AI/ML tools, to reduce rather than aggravate the inequities in our society.

## The Role of ML in the Practice of Medical Decision Making

All of these issues (and more) influence the acceptability of using ML to guide the decisions of practitioners, patients, and policy makers. Challenges related to design, parameterization, validation, and interpretability all influence the responsible adoption and consideration of ML outputs in medical decision making.

It is for this reason that *Medical Decision Making* has launched a call for original research related to the interface between AI driven by ML and medical decision making.^14^ The call identifies a number of critical issues related to the use of ML models in practice, including explainability, usability, management of algorithm bias, nonstatistical criteria for designing and selecting models, and the influence of these models on decisions in practice. We hope that the call encourages a robust conversation about the role of ML and its limitations in supporting health care transformation programs and improving patient support policies and practice.
